# Analysis of potential strategies for cadmium stress tolerance revealed by transcriptome analysis of upland cotton

**DOI:** 10.1038/s41598-018-36228-z

**Published:** 2019-01-14

**Authors:** Haodong Chen, Yujun Li, Xiongfeng Ma, Lishuang Guo, Yunxin He, Zhongying Ren, Zhengcheng Kuang, Xiling Zhang, Zhigang Zhang

**Affiliations:** 1 Cotton Sciences Research Institute of Hunan/ National Hybrid Cotton Research Promotion Center, Changde, Hunan 415101 China; 2 State Key Laboratory of Cotton Biology, Institute of Cotton Research, Chinese Academy of Agricultural Sciences, Anyang, Henan 455000 China

## Abstract

In recent years, heavy metal pollution has become a more serious global problem, and all countries are actively engaged in finding methods to remediate heavy metal-contaminated soil. We conducted transcriptome sequencing of the roots of cotton grown under three different cadmium concentrations, and analysed the potential strategies for coping with cadmium stress. Through Gene Ontology analysis, we found that most of the genes differentially regulated under cadmium stress were associated with catalytic activity and binding action, especially metal iron binding, and specific metabolic and cellular processes. The genes responsive to cadmium stress were mainly related to membrane and response to stimulus. The KEGG pathways enriched differentially expressed genes were associated with secondary metabolite production, Starch and sucrose metabolism, flavonoid biosynthesis, phenylalanina metalism and biosynthesis, in order to improve the activity of antioxidant system, repair systems and transport system and reduction of cadmium toxicity. There are three main mechanisms by which cotton responds to cadmium stress: thickening of physical barriers, oxidation resistance and detoxification complexation. Meanwhile, identified a potential cotton-specific stress response pathway involving brassinolide, and ethylene signaling pathways. Further investigation is needed to define the specific molecular mechanisms underlying cotton tolerance to cadmium stress. In this study potential coping strategies of cotton root under cadmium stress were revealed. Our findings can guide the selection of cotton breeds that absorb high levels of cadmium.

## Introduction

Cadmium pollution is a global environmental issue, and the development of modern industry and agriculture has led to more and more water and soil being polluted by cadmium every year^1–3^. Cadmium can accumulate for long periods of time inside animals and plants, affecting growth and development and posing a great danger to human health. The high tolerance of cotton to cadmium stands out from all extensively planted crops. Cotton is capable of absorbing a considerable amount of cadmium, and lower concentrations of cadmium has even been shown to promote development, productivity and fiber quality, and the effects of cadmium on a variety of physiological and biochemical characteristics as well as agronomic characteristics are very limited when the cadmium concentration is below 0.2 mmol. In addition, the cadmium content in cotton fibers, the main product of cotton, remains relatively low^4–6^. Therefore, cotton is a promising crop for treating cadmium-polluted soil.

Cadmium mainly causes damage to the plant by disrupting the balance of oxidation-reduction in cells. Specifically, cadmium accumulation leads to the production of a large quantity of reactive oxygen species (ROS) that oxidize membrane lipids, causing damage to the membrane system, affecting normal cellular functions and even leading to the collapse and death of the endomembrane system^7–9^. Previous studies have shown that there are three main cadmium resistance strategies in plants. The first strategy is the absorption of cadmium or isolation of cadmium inside plant. The second is alleviation of cadmium (Cd) toxicity and cadmium (Cd) removal through a series of chelating mechanisms. The third is the removal of the oxygen species (ROS) that accumulate during cadmium (Cd) stressPlants store cadmium at specific locations inside the plant and reduce the cadmium concentration in plant tissues that are metabolically active^10,11^. For example, hyper-accumulators like *Brassica juncea* store absorbed cadmium in the epidermis and epidermic hair cells, which are physiologically less active in order to alleviate the toxicity of cadmium (Cd) in other tissues. Cadmium absorbed via the symplast pathway in *Oryza sativa* accumulates in the vacuoles of root cortex cells, so that the number of cadmium ions entering the microtubules cells is reduced. When tobacco is exposed to cadmium stress, a large quantity of cadmium accumulates in the root cytoderm, which prevents cadmium ions from entering microtubules cell via the apoplast pathway. In addition, some cadmium ions bind to the active groups of cellulose and lignin, such as carboxyl and hydroxyl groups, reducing the quantity of cadmium that enters the protoplasm, and alleviating damage to the plant^12–14^.

Strategies for removal of cadmium that has accumulated inside cells mainly involve the induction of phytochelatin, metallothionein and relevant transport proteins that ultimately remove chelates out of the cell. The Arabidopsis thaliana mutants, cad1, cad2 and vtc1, which lack a phytochelatin (PC) synthesis system, have increased sensitivity to cadmium^15–17^. Overexpression of CAD1 in *Brassica juncea* increases its cadmium resistance, and overexpressing wheat phytochelatin (PC) synthase gene in tobacco also show increased cadmium resistance^18,19^. metallothionein (MT) genes such as CeMT2b, SaMT2 and TaMT3 overexpressing in *Nicotiana tabacum L* show even higher cadmium resistance^20–22^. The anti-oxidative mechanism of plants against the accumulation of oxygen species (ROS) is now clear, and includes the up-regulation of antioxidant enzymes such as superoxide superoxide dismutase, catalase, peroxidase, ascorbate peroxidase, and glutathione S-transferase, the improvement of the activity of anti-oxidative system, and the synthesis of a large quantity of anti-oxidative substances that accelerate the removal of oxygen species (ROS)^23^.

Even though the mechanism of cadmium resistance is known for some plants, the specific factors that affect the growth of cotton under cadmium stress still remain unclear. In this study, we investigated the cadmium resistance mechanism of cotton by analyzing the transcriptome data of cotton roots under cadmium stress and using existing studies as references. Our findings will provide insight that guide further studies of the cadmium resistance signaling pathways in cotton, as well as breeding of cadmium-resistant cotton species.

## Results and Discussion

It is known that heavy metal ions enter plants via the symplast pathway and the apoplast pathway, and the root is the primary site of heavy metal absorption. Because the root is the first tissue to be poisoned by cadmium, it is prudent to investigate the mechanisms for heavy metal tolerance by analyzing plant root. Hence, we have conducted transcriptomic analysis of roots treated with different concentrations of cadmium.

### Illumina Solexa paired-end RNA sequencing and read mapping

**Figure 1:**
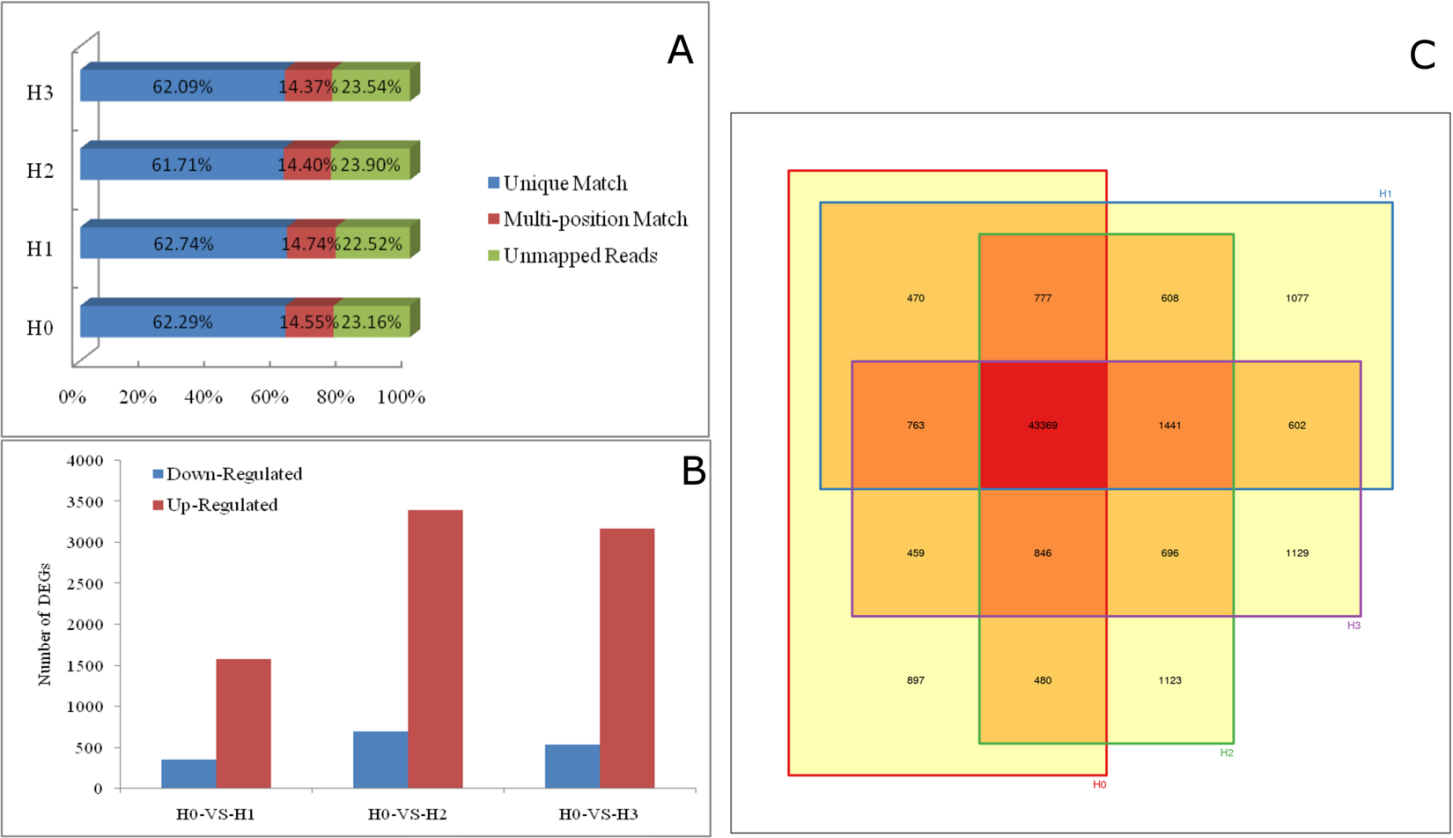
RNA-sequencing results and differentially expressed genes. (**A**) The percentage of RNA-sequencing reads that mapped to the reference. (**B**) The number of significantly up-regulated (red) and down-regulated (blue) genes between cotton roots treated with three cadmium concentrations (H1, H2 and H3) and the untreated control (H0). (**C**) Venn diagram illustrating the overlap in transcripts between samples.

Seedlings of the upland cotton cultivar C184 were exposed to three different concentrations of cadmium using a hydroponics system. RNA from the root tissues of the control group (H0) and cadmium-treated groups (H1, H2 and H3) was sequenced on the Illumina HiSeqTM 2000 platform. The clean reads from the four samples accounted for nearly 93% of the raw data. The clean reads were mapped to the reference genome with Gossypium hirsutum TM-1 (http://mascotton.njau.du.cn), and in all four samples nearly 76% matched reference sequences. of these mapped reads, about 62% were unique matches, and 14%~15% mapped to multiple locations (Fig. 1A). A total of 48061, 49107, 49340 and 49305 transcripts were detected in H0, H1, H2 and H3, respectively, and the number transcripts iidentified in all four samples was 43,369 (Fig. 1B). The number of differentially expressed gene in all cadmium-treated groups compared with the control group was 1441, and the number of genes with log_2_^(Relative value)^ change in expression equal to or greater than 1 or equal or less than −1 was 1151 (Fig. 1C). The number of differentially expressed genes identified by comparing H1 and H0 was 1908, among which the expression of 1565 genes were up-regulated in H1. The number of differentially expressed genes between H2 and H0 was 4075, of which 3165 were up-regulated in H3. These differentially expressed genes in H1, H2 and H3 were mapped to 115, 119, and 120 KEGG categories respectively.

### Characterization of differentially expressed genes

**Figure 2:**
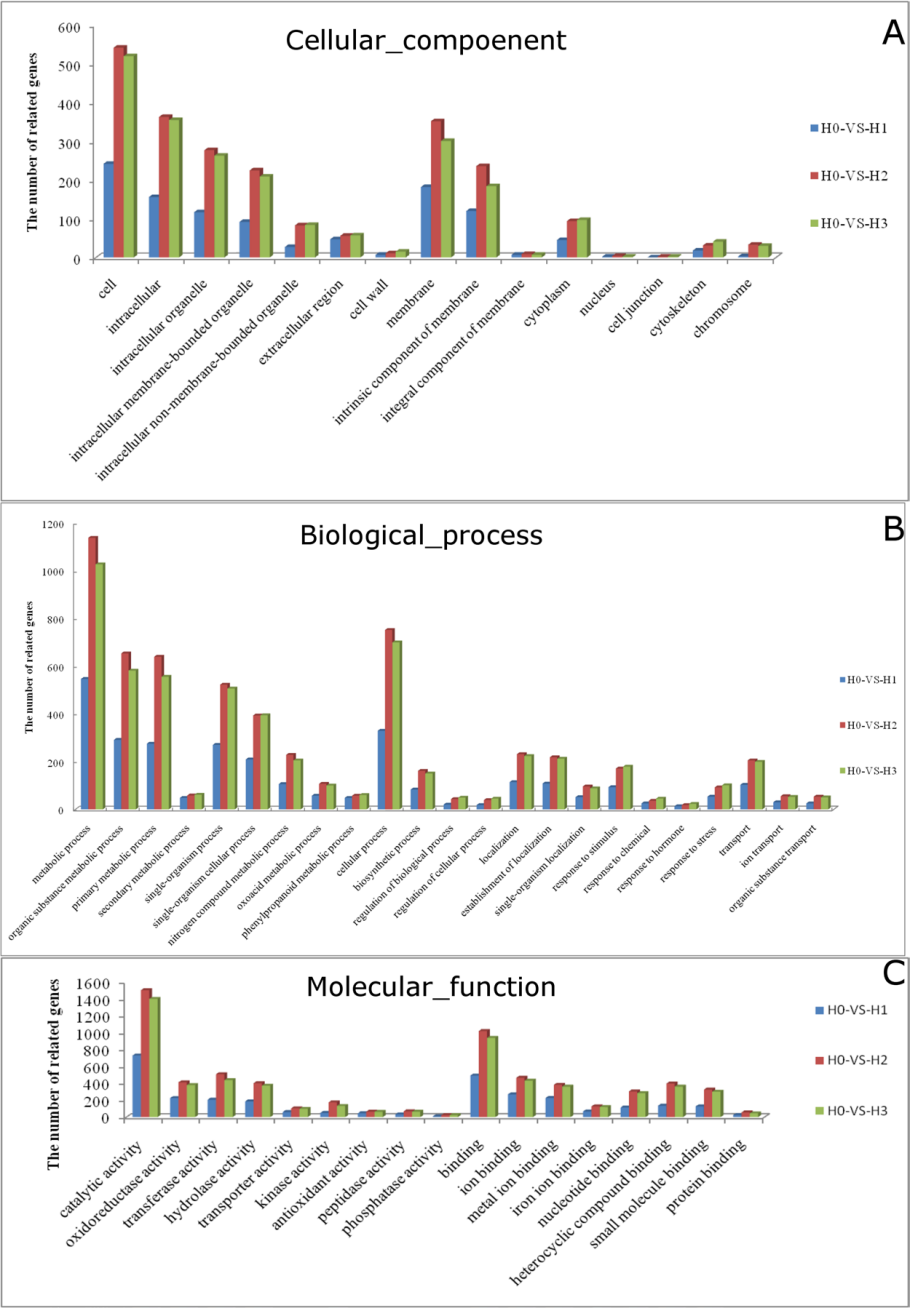
Gene ontology classification of cotton root genes differentially expressed under different Cd concentrations. (**A**) cellular component (**B**) biological process and (**C**) molecular function.

We used gene ontology annotations to classify the differentially expressed genes based on function and found that for each of the three cadmium-treated samples (H1, H2 and H3), the proportions of differentially expressed genes compared with H0 in gene ontology (GO) functional cluster were basically consistent, but there were differences in specific clusters. gene ontology (GO) annotations can be grouped into three broad categories: molecular function, cellular composition and biological process. With respect to cellular composition, the proportion of differentially expressed genes that function in intracellular regions is relatively larger, and the ratio of intracellular to extracellular differentially expressed genes increases as cadmium (Cd) concentration increases. The proportion of genes related to intracellular membranous organelles accounts for a considerable portion(Fig. 2A). In addition, the number of differentially expressed genes related to cytomembrane is far greater than the number related to other cellular components, indicating that cadmium (Cd) stress may affects the membrane system of root cells, especially the combination of membrane system. The effect of cadmium (Cd) stress on extracellular structures is limited, for example the cell junctions.

With respect to biological process (Fig. 2B), the genes differentially expressed in response to cadmium (Cd) stress are mainly related to primary metabolism, with a smaller related to secondary metabolism. The metabolism of nitrogen-containing compounds, the synthesis of phenylpropanoids, and the synthesis of ketonic acid are three major biological processes that have a major impact on cadmium stress^24,25^. From the perspective of the cellular processes, the effects of cadmium stress are mainly taking place on the localization, response to stimulus, transport, while biological synthesis is a process that accounts for a considerable proportion. There is also a large proportion of differentially expressed genes that are related to the localization of substances and organelles, suggesting that cadmium stress increases the frequency of synthesis and transport of substances in root cells. And a severe change occur both in intracellular and extracellular environment while various response mechanism of cells start activating. Stress signaling under cadmium mainly include response to chemical and response to hormone process. Meanwhile, similarities of the stress processes when being treated with biological threat such as the invasion of pathogen are also demonstrated. With respect to substance transport, differentially expressed genes are mainly related to the transport of ions and organic substances, indicating that cadmium stress may disrupt ionic balance, and a dramatic increase in transport of substances, such as secondary metabolites. A number of studies have shown that ions, especially metal ion transporters do in fact play a key role in the absorption and accumulation of heavy metals in plants.

With respect to molecular functions, a considerable proportion of differentially expressed genes are related to catalytic activity, oxidoreductase activity and transferase activity (Fig. 2C), which is consistent with previous analysis. In addition, we found that a large number of differentially expressed genes are related to antioxidant enzyme activities and kinase activity suggesting that kinases function in cadmium stress signaling pathways. In the process of cell junction, the junction of metal ions, heterocyclic compounds and small molecular substances are of higher number of differentially expressed genes, indicating that metal ions, especially iron ions have more signal pathways of engagement in the reaction of cadmium stress, which is consistent with existing studies, wu study showed that maintaining high iron content in shoot under cadmium (Cd) exposure could alleviate the cadmium (Cd) toxicity. Meanwhile, the hybrid compounds and small molecular substances produced during metabolism are maybe play a important role in cadmium stress signaling pathways.

**Figure 3:**
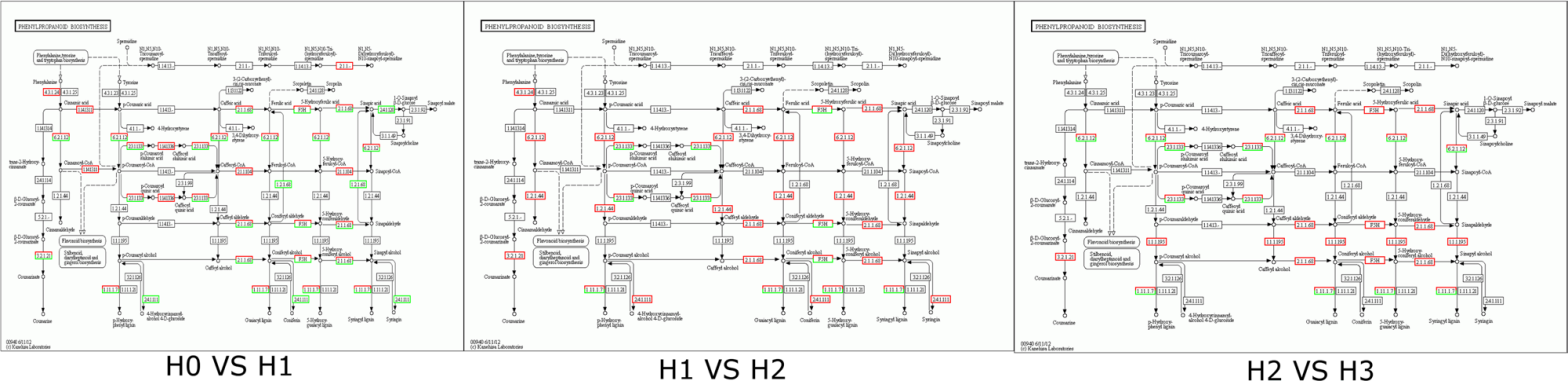
Impact of cadmium stress on phenylpropanoid biosynthesis (https://www.kegg.jp/ dbget-bin/www_bget?map00940)under different Cd concentrations.

Analysis of the KEGG metabolic pathways enriched in genes differentially expressed in response to cadmium stress indicates that nearly 30% are involved in the synthesis of secondary metabolite pathways, including phenylpropanoid synthesis, phenylalanine metabolism, and flavonoid synthesis. Phenylpropanoid synthesis (https://www.kegg.jp/dbget-bin/www_bget?map00940) and PHE metabolism are closely related to the synthesis of lignin as well as plant disease responses (Fig. 3). Flavonoid metabolites product mainly include pigments, antioxidants, and small signaling molecules, which are closely related to plant stress resistance, indicating that in cotton the mechanisms for responding to cadmium and biological stress are similar. Cadmium stress leads to dramatic increase in metabolic activities on a cellular level, and also the increase of metabolites with hydrolytic activities.

### Coping with cadmium stress by forming a physical barrier

Heavy metal tolerance of plants includes two aspects: one is the removal of heavy metals absorbed by the plant or impeding their transport withinin the plant; the other is detoxification by a variety of mechanisms, such as the binding of heavy metal to the cytoderm, which is then removed from the cell through the formation of organic acid and protein complexes. To further understand the mechanism for cadmium tolerance in cotton, we conducted a comprehensive analysis of 1151 genes differentially expressed in all three cadmium stress samples.

Cellulose is the main component of the plant cytoderm. Of the 1151 differentially expressed genes, the expression levels of 19 genes related to the synthesis of cellulose increased significantly; all of these genes are homologs of cellulose synthase (K10999) (Fig. 2). cellulose synthase A7 (CotAD_38396), cellulose synthase A8 (CotAD_23453), CotAD_69280), and cellulose synthase A4 (CotAD_57824, CotAD_54812) are correlated with the synthesis of the cellulose of primary cytoderm^26^, and cellulose synthase A9 (CotAD_62834, CotAD_51434), cellulose synthase A10 (CotAD_72572), CSLC (CotAD_58043, CotAD_13312) participates in the synthesis of the cellulose of secondary cytoderm^27–29^ (Table 1). In a previous study by Chen *et al*. on the effects of cadmium (Cd) on different components of willow cytoderm components, cellulose was found to strengthen by the absorption of cadmium (Cd) by cytoderm^30^. The increase in cellulose will in turn increase the absorption of cadmium (Cd) ions by cytoderm, reducing the number of cadmium (Cd) ions between cells, and consequently reducing the introduction of cadmium (Cd) into cells via the apoplast pathway, and thus alleviating cadmium (Cd) toxicity. We found that a large number of pectin methylesterase inhibitor genes and polygalacturonase-inhibiting protein genes were also up-regulated, and the hydrolysis process of pectin that is another cell wall component was inhibited. Previous studies of wheat, chilies, and kiwis have shown that overexpression of methylesterase inhibitor genes (PMEI) may lead to increased methyl esterification of pectin in the cytoderm, while suppressing the activities of pectin methylesterase and polygalacturonase, which are related to pectin hydrolysis^31–33^. Thus, the cytoderm is thickened, enhancing plant resistance to biological and abiotic stress. In addition, decreased methylesterase activity leads to the reduction of the carboxy l produced during pectin de-esterification in the cytoderm of peripheral cells, consequently decreasing the of absorption of positive heavy metal ions, such as cadmium (Cd) by root cells, as well as the amount of heavy metals absorbed by plants^34^.

**Table 1:** Differential expression genes in cellulose synthesis process under cadmium stress in cotton root.

Gene and Blast	log2Ratio(H0-VS-H1)	log2Ratio(H0-VS-H2)	log2Ratio(H0-VS-H3)
**The cellulose synthesis genes of primary cell wall**			
CotAD_38396 CESA7	1.557482	1.713838	1.983378
CotAD_23453 CESA8	1.591243	2.027124	1.774615
CotAD_69280 CESA8	1.058526	1.281549	1.351638
CotAD_57824 Cellulose synthase A4	2.456065	2.864345	2.462791
CotAD_54812 Cellulose synthase A4	3.571157	3.405992	3.560715
CotAD_00575 Cellulose synthase A1	1.837912	2.082523	2.219553
CotAD_10480 Cellulose-synthase-like C5	1.98907	1.144908	1.882366
CotAD_56775 Cellulose-synthase-like C5	2.391528	1.295149	1.885731
CotAD_05132 Cellulose synthase-like B3	−3.263034	−1.607683	−2.29956
CotAD_46937 Cellulose synthase-like E1	−0.293683	−1.034949	−0.138555
CotAD_53925 Cellulose synthase like G3	−0.722466	−1.552541	−2.956931
CotAD_19832 Cellulose synthase like G3	−11.049849	−0.88243	−0.568049
**The cellulose synthesis genes of Secondary cell wall**			
CotAD_62834 CESA9	1.69329	1.822638	2.359511
CotAD_51434 CESA9	1.938548	2.234141	2.487153
CotAD_72572 CESA10	1.501478	2.286739	2.522806
CotAD_58043 Cellulose-synthase-like C12	1.460226	1.528496	2.233591
CotAD_13312 Cellulose-synthase-like C4	1.2935	2.003514	2.295651
**Other related cellulose synthesis genes**			
CotAD_48408 Cellulose synthase	1.419384	1.639206	2.051312
CotAD_51651 Cellulose synthase	1.864864	2.215985	2.176928
CotAD_61788 Cellulose synthase 2-Dt	1.661831	2.121881	2.292562
CotAD_63213 Cellulose synthase family protein	1.113962	1.30248	1.651
CotAD_10636 Cellulose synthase like G2	1.379658	2.02403	1.714423
CotAD_10635 Cellulose synthase like G2	1.23078	2.061952	1.665133
CotAD_59099 Beta tubulin	3.04459	3.585502	5.046353
CotAD_21797 ATP binding microtubule motor family protein	1.429138	1.463174	1.78061
**Pectin hydrolysis related genes**			
CotAD_11961 Pectin methylesterase inhibitor superfamily	4.137504	2.918386	3.996389
CotAD_01805 Pectin methylesterase inhibitor superfamily	2.632695	3.375199	3.27894
CotAD_22628 Pectin methylesterase inhibitor superfamily	2.650687	3.059871	3.184791
CotAD_45983 Pectin methylesterase inhibitor superfamily	3.063326	4.725126	3.411565
CotAD_59548 Pectin methylesterase inhibitor superfamily	2.881072	3.27894	3.441269
CotAD_71768 Pectin methylesterase inhibitor superfamily	2.849094	3.333737	3.500173
CotAD_50138 Pectin methylesterase inhibitor superfamily	1.357719	2.728178	1.879046
CotAD_70731 Pectin methylesterase inhibitor superfamily	1.765261	3.091386	1.794206
CotAD_32591 Pectin methylesterase inhibitor superfamily	2.189825	2.369234	2.254241
CotAD_76273 Pectin methylesterase inhibitor superfamily	1.72259	2.10728	1.578135
CotAD_45985 Pectin methylesterase inhibitor superfamily	−1.515442	−2.166455	−1.438093
CotAD_28069 Pectin methylesterase inhibitor superfamily	−0.630113	−0.462516	−1.613652
CotAD_63272 Pectin methylesterase inhibitor superfamily	−0.734779	−0.81509	−1.655598
CotAD_06244 Polygalacturonase-inhibiting protein	1.22272	2.802103	1.630121
CotAD_07327 Polygalacturonase-inhibiting protein	1.540627	2.810026	1.674442
CotAD_17205 Polygalacturonase-inhibiting protein	−1.179787	−0.96138	−1.171961

**Figure 4:**
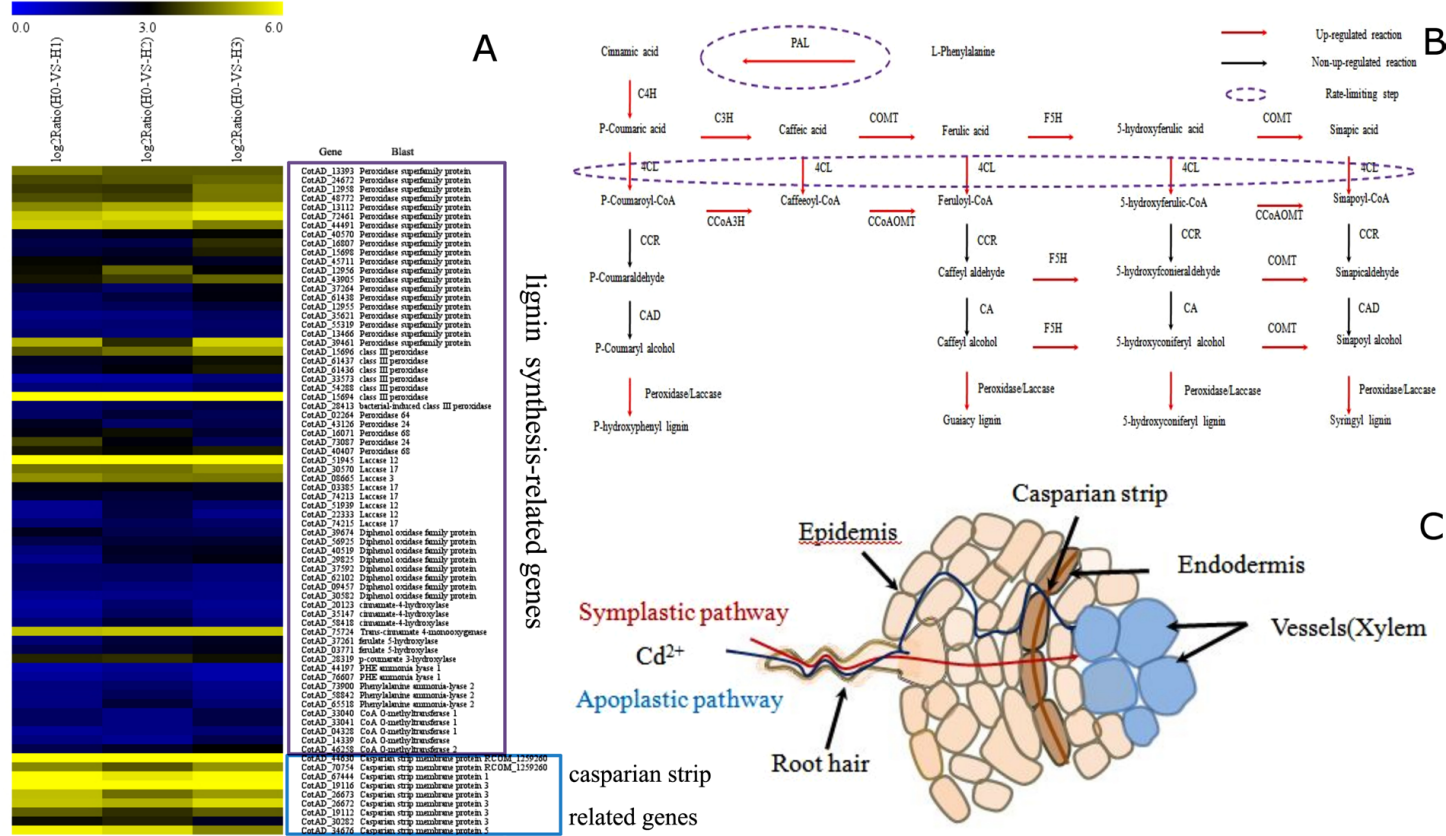
The physical barrier strategy for cadmium tolerance in cotton. (**A**) A heat map illustrating the differential expression of genes related to the formation of a physical barrier under Cd stress. x axis is log_2_^x^ value of relative expression level. (**B**) Lignin biosynthetic pathway genes differentially expressed under cadmium stress. (C) A schematic diagram of the obstruction of cadmium ion transport by the Casparian strip in cotton roots.

Cadmium stress led to a significant increase in the expression of genes involved in the synthesis of another cytoderm component, lignin^35^. Enzymes involved in the general lignin synthesis pathways were all up-regulated, and the expression levels of genes encoding the rate-limiting enzyme of lignin synthesis, Phenylalanine ammonia lyase, including CotAD_73900, CotAD_58842, CotAD_65518, doubled^36^. The expression levels of genes encoding another rate-limiting enzyme, 4-coumarate: CoA ligase, including CotAD_20123, CotAD_35147, CotAD_58418, were also significantly increased^37^. The expression levels of other genes, such as cinnamate-4-hydroxylase, ferulate 5-hydroxylase, p-coumarate 3-hydroxylase and CoA O-methyltransferase, which are involved in other lignin synthesis processes^38^, also increased to different extents (Fig. 4A,B). Lignin is the main component of secondary cytoderm, and cadmium stress increases lignin synthesis in cotton roots, the thickening of secondary cytoderm, and hence the reduction of the absorption of cadmium (Cd) by the root system, and the improvement of cadmium (Cd) tolerance in cotton. The cotton similar *Matricaria chamomilla* under Cu and cadmium (Cd) stress, lignin is found to accumulate in the cytoderm, forming a barrier against the absorption of heavy metal, and the cadmium (Cd) ions bind to acidic functional groups of lectin on the cell surface and to phenolic aldehyde led reduce cadmium (Cd) poisoning^39^. Therefore, some genes related to lignin synthesis are upregulated suggest that lignin plays an important role in cadmium tolerance in cotton, and its absorption of cadmium may reduce the cadmium (Cd) that enters root cells, consequently alleviating effects of cadmium (Cd) on the growth and development of the cotton root system.

The casparian strip, which consists of proteins, lignin, suberinite and cellulose, is another important barrier that prevents positive metal ions such as cadmium (Cd) from entering the cortex via the apoplast pathway (Fig. 4C). Among them, casparianstrip membrane domain proteins (CASPs) are the key signals for the initiation and localization of the casparian strip (CASP), which as casparian strip (CASP) polymer composites are firmly stuck to plasma membranes. And the casparian strip (CASP) formed by the accumulation of substances such as lignin and suberinite, impedes the transport of positive ions. For example, previous studies have found that calcium and lanthanum ions are incapable of penetrating casparian strip (CASP) of corn roots^40,41^. Seregin *et al*. found that the concentration of cadmium (Cd) ions within the root cortex of corn seedlings was significantly lower than that outside casparian strip, indicating that casparian strip (CASP) did impede the transport of cadmium ions^42^. As a result, corn seedlings are tolerate certain cadmium concentrations. Here we found that the expression level of casparian strip (CASP) genes, including one *CASP1*, five *CASP3* and one *CASP5* homologs, significantly increased upon cadmium treatment, indicating that the casparian strip (CASP) in the cotton root endodermis becomes broader, wider and deeper in response to cadmium stress. The expression levels of genes encoding specific enzymes involved in the synthesis of fatty acids and extension of fatty acid chains also increased. These results together with the increased expression levels of lignin and cellulose synthesis genes, are consistent with thickening of casparian strip of roots and the impediment of ion transport.

In conclusion, in cotton root cells cadmium stress leads to the thickening of the cytoderm, the thickening and broadening of casparian strip, and blocking of the transport of cadmium ions from both apoplast and symplast pathways. This excludes cadmium ions from the cortex and alleviates the effects of cadmium toxicity.

### Oxidation resistance and complexation detoxification

**Figure 5:**
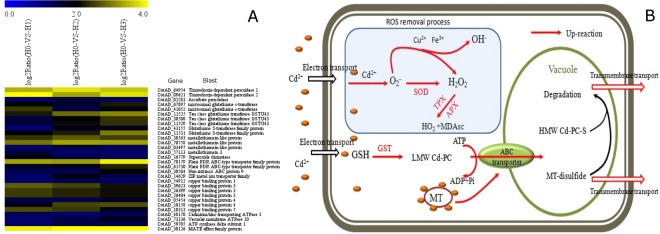
The oxidation resistance and complexation detoxification strategies for cadmium tolerance in cotton. (**A**) A heat map illustrating the differential expression of genes related to oxidation resistance and complexation detoxification under Cd stress. x axis is log_2_^x^ value of relative expression level. (**B**) A diagram illustrating the functions of differentially expressed genes in oxidation resistance and complexation detoxification

**Figure 6:**
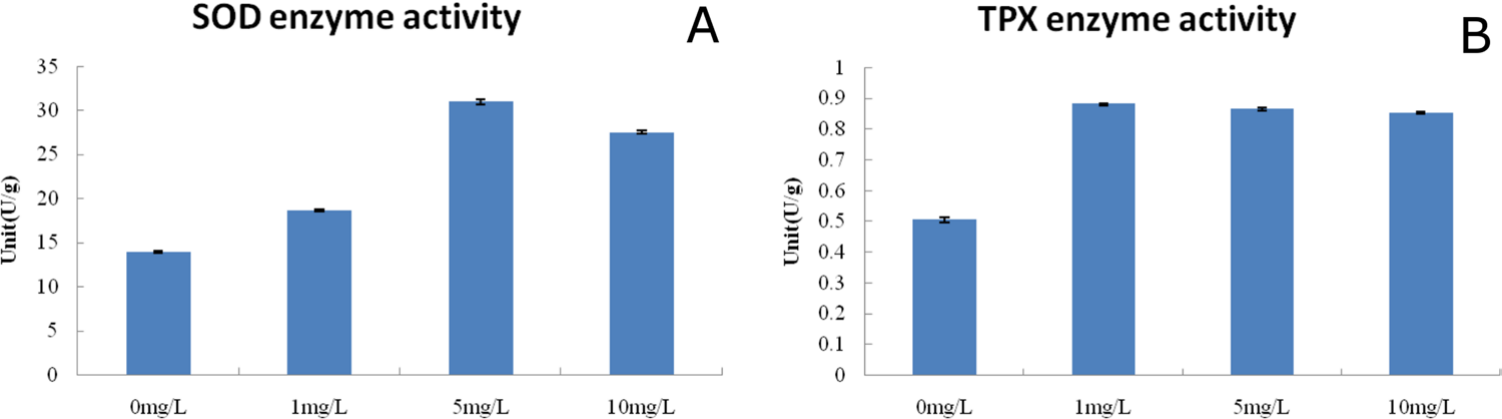
The determination of Superoxide Dismutase (SOD) and thioredoxin peroxidase (TPx) enzyme activity under cadmium stress in cotton roots.

Studies have shown that as soon as cadmium (Cd) ions enter plant cells, oxygen species (ROS) begin to accumulate, oxygen species (ROS) which results in a series of physiological and metabolic disorders. Analysis of the cadmium stress transcriptome data revealed that the accumulation of cadmium (Cd) ions and oxygen radicals in cotton root tissue may induce stress responses, including oxidation resistance and heavy metal complexation (Fig. 5A,B). The expression level of the key enzyme involved in oxidation resistance, superoxide (SOD), was upregulated. Cu^2+^ transport proteins were also up-regulated, indicating a potential increase in Cu^2+^ concentration in cells, and hence the potential increase in the activities of several antioxidative metalloenzymes, such as superoxide (SOD), laccase and polyphenol oxidase^43,44^. Thioredoxin peroxidase and ascorbate peroxidase (APx), which are involved in the decomposition of H_2_O_2_, a secondary product of oxygen species (ROS), were also significantly up-regulated. The activity of superoxide (SOD), Thioredoxin peroxidase (TPx) and ascorbate peroxidase (APx) remove oxygen species (ROS) and prevents cell metabolic disorders. The increased activity of superoxide (SOD) and Thioredoxin peroxidase (TPx) in the cotton root under cadmium stress was verified by enzyme assays (Fig. 6).

cadmium (Cd) ions already located inside cells are detoxified by heavy metal chelating agents such as phytochelatin (PC), glutathione and metallothionein (MT). While the expression quantity of PDR-type ABC transport protein (CotAD_70179, CotAD_61730), MATE transport protein gene (CotAD_30126) and ATP synthetase involved in the transport of heavy metal chelates also increased for cadmium (Cd) detoxification. When cotton root tissues are treated with cadmium, glutathione (GSH) transferase (GST) catalyzes conjugation of glutathione (GSH) to heavy metals. Cotton GST as well multiple metallothionein (MT) genes were up-regulated by cadmium stress. The expression level of genes encoding PDR-type ABC transport protein (CotAD_70179, CotAD_61730) and MATE transport protein (CotAD_30126), which are involved in the transport of heavy metal chelates, and ATP synthetase, which supplies engery needed for transport, were also upregulated. Previous studies by Kim *et al*. and Ogawa *et al*. showed that the expression of *Oryza sativa* and *A. thaliana* PDR genes and MATE transport protein genes were induced by metal ions; among them, PRD8 was shown to be involved in the transport of Cd^2+^ or cadmium (Cd) chelates in arabidopsis^45–47^.

### Effect of cadmium on the development of the cotton root

**Figure 7:**
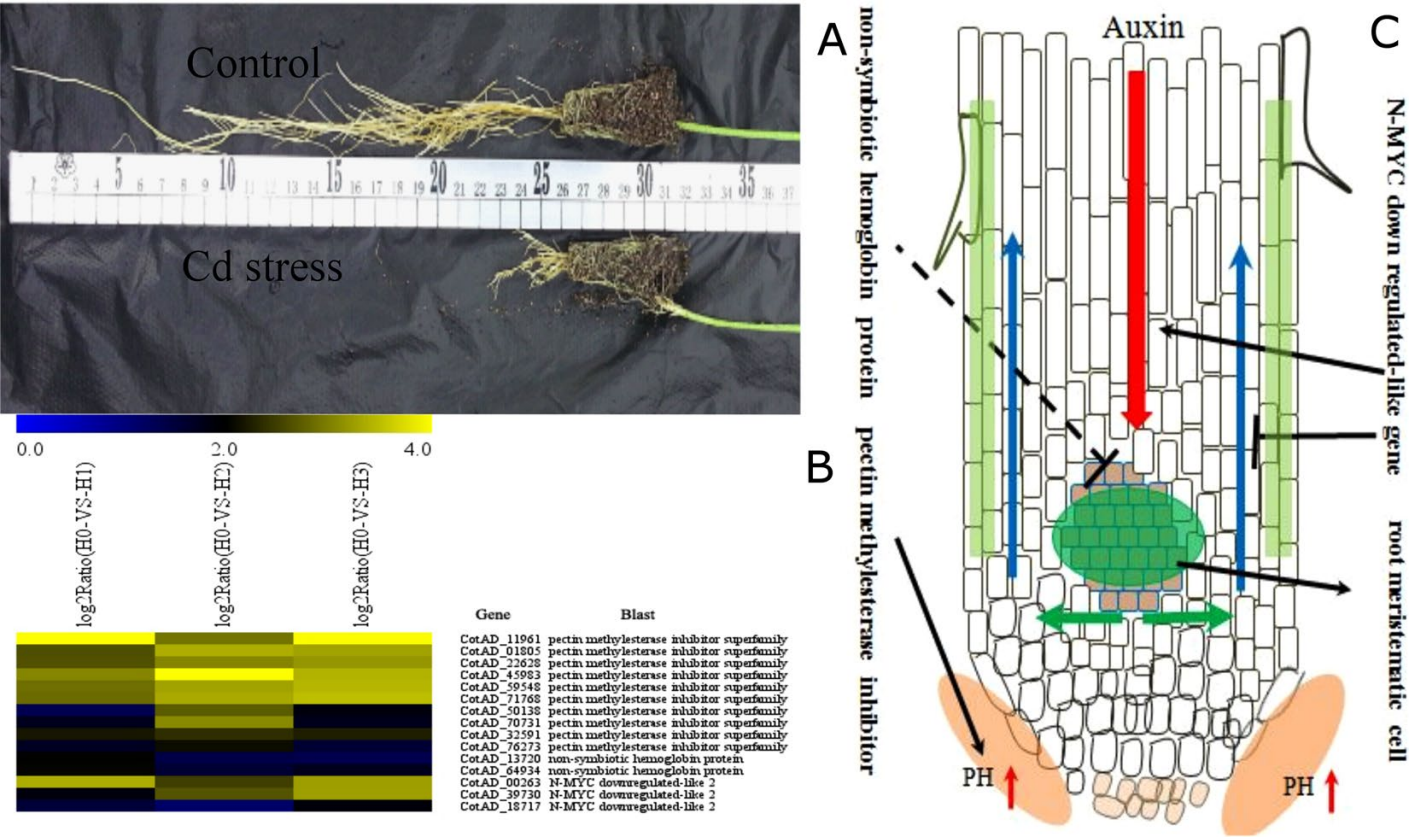
The influence of cadmium stress on cotton root development. (**A**) The root systems of 30 day old control and Cd stress (5 mg/L) cotton treated seedlings. x axis is log_2_^x^ value of relative expression level. (**B**) Genes A heat map illustrating the differential expression of genes related to root development under cadmium stress. (**C**) Diagram illustrating the regulation of root growth under cadmium stress in cotton.

Exposure of cotton grown in solution to cadmium stress significantly suppressed root development, especially the development of primary roots (Fig. 7A). Similar phenomena have been found by Liu in cotton cadmium stress experiment^48^. We found that three types of genes related to root development, methylesterase inhibitor (PMEI), nonsymbiotic hemoglobin, and N-MYC downregulated-like 2, were differentially expressed upon cadmium treatment (Fig. 7B). The up-regulation of methylesterase inhibitor (PMEI) genes suppresses the de-esterification of pectin, which also reduces the amount of oxygen ions produced during the de-esterification, leading to an increase in the pH in the root. According to the acid curvature theory, this increase in pH will inevitably affect the extension of root tip cells, consequently suppressing root growth^49^ and potentially explains why cadmium suppresses cotton root development. Expression of nonsymbiotic hemoglobin (nsHb) is an important strategy for stress resistance. Previous studies by Igamberdiev *et al*. and Yang *et al*. showed that overexpression of nonsymbiotic hemoglobin (nsHb) genes increase plant antioxidase activity and strengthens resistance against oxidative stress^50,51^. Moreover, Parent *et al*. discovered that the nonsymbiotic hemoglobin (nsHb) gene was highly expressed specifically in the protoderm and xylem of root tissues^52^. Therefore, the up-regulation of cotton nonsymbiotic hemoglobin (nsHb) by cadmium stress may be another factor suppressing root development. Studies have shown that the downregulated-like 2 (NDL2) gene functions with heterotrimeric G-protein to regulate auxin transport within plants, and Yashwanti *et al*. verified that NDL increases the transport of basipetal auxin, while suppressing the transport of auxin towards the top of the plant. Hence, the up-regulation of downregulated-like 2 (NDL2) by cadmium stress may lead to accumulation of auxin in root meristems, leading to impeded development of primary roots, such as observed under cadmium stress^53^. Taken together, our finding that methylesterase inhibitor (PMEI), nonsymbiotic hemoglobin (nsHb) and downregulated-like 2 (NDL2) genes are upregulated by cadmium stress, is consistent with the suppression of root development (Fig. 7C).

### Differentially expressed transcription factors under cadmium stress in cotton roots

We found that 28 transcription factors were differentially expressed in all three cadmium-treated cotton samples compared with the control group. Of these, 20 were upregulated including, MYB, zinc finger (GATA-type, CCCH-type, and C2H2-type), leucine zipper and NAC transcription factors (Table 2). Based on studies in *A. thaliana* the homologs of these transcription factors with known functions are mainly involved in the transduction of light signals, the synthesis of brassinolide (BR), brassinolide (BR) signal transduction, the synthesis of the secondary cytoderm, and ethylene signal transduction.

**Table 2:** Differential expression transcription factors under cadmium stress in cotton root.

Gene ID	log2Ratio(H0-VS-H1)	log2Ratio(H0-VS-H2)	log2Ratio(H0-VS-H3)	Blast and description	The function of homologous gene in other species
**Up-regulated transcripts**					
CotAD_02717	5.229531	4.714754	5.49896	Myb domain protein 18[Theobroma cacao]	Positive regulator of the phyA photoresponse
CotAD_23906	3.424715	3.49136	4.690159	Myb domain protein 55 [Theobroma cacao]	involved in Brassinosteroid homeostasis and growth responses
CotAD_19868	2.710493	2.890771	3.460425	Myb domain protein 40 [Theobroma cacao]	function unknown
CotAD_76285	2.297773	2.479582	2.641895	Myb domain protein 40 [Theobroma cacao]	function unknown
CotAD_50930	1.830316	1.64293	1.60539	Myb-like HTH transcriptional regulator protein [Theobroma cacao]	function unknown
CotAD_62038	1.674672	2.007526	2.324341	MYb transcription factor [Gossypium hirsutum]	involved in the response to UV-B
CotAD_39114	1.063645	1.621674	2.095315	Myb domain protein 55 [Theobroma cacao]	involved in Brassinosteroid homeostasis and growth responses
CotAD_48495	2.645083	2.98263	3.303392	GATA transcription factor 9 [Theobroma cacao]	function unknown
CotAD_23031	2.341828	2.160992	2.989946	GATA transcription factor 9 [Theobroma cacao]	function unknown
CotAD_75922	1.556025	1.49199	2.442026	GATA transcription factor 9 [Theobroma cacao]	function unknown
CotAD_59298	1.369646	1.944693	2.293563	GATA transcription factor 2 [Theobroma cacao]	positive regulator of photomorphogenesis
CotAD_36519	1.357823	2.199698	1.54739	GATA zinc finger protein regulating nitrogen assimilation [Theobroma cacao]	function unknown
CotAD_55882	1	1.053503	1.195975	GATA zinc finger protein regulating nitrogen assimilation [Theobroma cacao]	function unknown
CotAD_38727	2.49114	3.372333	2.093109	Zinc finger protein [Theobroma cacao]	involved in secondary wall biosynthesis
CotAD_30228	1.973305	2.385891	2.909453	C2H2-type zinc finger family protein[Theobroma cacao]	function unknown
CotAD_53425	3.342392	3.1836	3.336963	Basic-leucine zipper transcription factor family protein [Theobroma cacao]	involved in lipid metabolism or cellular transpor
CotAD_16854	1.905141	2.242857	2.20543	Basic-leucine zipper transcription factor family protein [Theobroma cacao]	involved in lipid metabolism or cellular transpor
CotAD_21422	1.999432	1.737874	1.377638	Integrase-type DNA-binding superfamily protein[Theobroma cacao]	involved in ethylene signaling pathways
CotAD_11710	1.654185	1.162601	2.048023	ethylene-responsive element-binding protein 5 [Gossypium barbadense]	involved in ethylene signaling pathways
CotAD_61187	1.843845	2.563242	1.513578	LOB domain-containing protein 4	function unknown
**Dn-regulated transcripts**					
CotAD_13693	−2.066401	−2.447005	−3.066401	Basic helix-loop-helix (bHLH) DNA-binding superfamily protein [Theobroma cacao]	involved in iron homeostasis
CotAD_55579	−2.024977	−1.152438	−3.169925	Basic helix-loop-helix (bHLH) DNA-binding superfamily protein [Theobroma cacao]	involved in iron homeostasis
CotAD_12483	−1.683411	−1.834735	−2.361483	Basic helix-loop-helix (bHLH) DNA-binding superfamily protein [Theobroma cacao]	involved in Interactions between Ethylene and Auxin
CotAD_54397	−1.374919	−1.533229	−1.290792	Basic helix-loop-helix (bHLH) DNA-binding superfamily protein [Theobroma cacao]	involved in Interactions between Ethylene and Auxin
CotAD_08994	−1.201244	−1.241635	−1.704707	Basic helix-loop-helix (bHLH) DNA-binding superfamily protein [Theobroma cacao]	involved in salicylic-dependent defense signaling response
CotAD_50063	−1.613672	−2.009292	−1.449406	NAC domain protein 9 [Gossypium hirsutum]	function unknown
CotAD_73593	−1.210922	−1.524039	−1.079658	NAC domain protein 9 [Gossypium hirsutum]	function unknown
CotAD_70812	−1.214829	−2.034157	−1.538036	Myb-like transcription factor family protein [Theobroma cacao]	function unknown

The transduction of light signals, synthesis of brassinolide (BR) and brassinolide (BR) signal transduction pathways are involved in plant photomorphogenesis. Processes such as brassinolide (BR) synthesis, synthesis of secondary cytoderm and ethylene signal response are closely related to the resistance of plants against abiotic stress. The *A. thaliana* homolog of CotAD_02717, ATMYB18, was shown to be a transcriptional activator involved in regulating the response to phytochrome signals, especially under low red light and far red light^54^. AtMYB4 was shown to be involved in the response to drought, high salt and ultraviolet radiation. In addition, Silvia *et al*. discovered that AtMYB4 is involved in the synthesis of flavonol, and the up-regulation of AtMYB4 leads to increased stress resistance^55–57^. So, the up-regulation of the expression of a large number of genes involved in phenylpropanoid and flavonoid synthesis pathways is consistent with increased resistance against cadmium stress in cotton. Interestingly, two types of genes that are involved in ethylene response are also related to stress resistance and tissue development. First, the *A. thaliana* homolog of CotAD_21422 and CotAD_11710, which are ethylene response factors, functions in oxidative stress and osmotic pressure signal transduction pathways^58,59^. The up-regulated expression of ethylene response genes suggests that the improved resistance to cadmium (Cd) in cotton is mediated via ethylene response pathways. Second, GATA2 and MYB55 have been reported to be key genes connecting the responses to brassinolide (BR) and developmental signals, and the expression levels of both genes were repressed by brassinolide (BR) though the BR-activated transcription factor BZR1^60,61^. Therefore, the increased expression of GATA2 and MYB55 suggests that the accumulation of brassinolide (BR) is decreased under cadmium (Cd) stress in cotton, and less brassinolide (BR) in root cells would impede root growth, leading to a shorter taproot. Other differentially expression transcription factors are predicted to be involved in cell wall synthesis, the metabolism and cellular transport of ester compounds and iron ion balance in the cell; all of these functions are associated with plant stress resistance. Thus, it indicates that the brassinolide (BR) signaling pathway activated in response to cadmium (Cd) stress in *A. thaliana* and cotton. The functions of other differentially expressed transcription factors still remain unclear and need to be further studied.

### Quantitative RT-PCR validation

**Figure 8:**
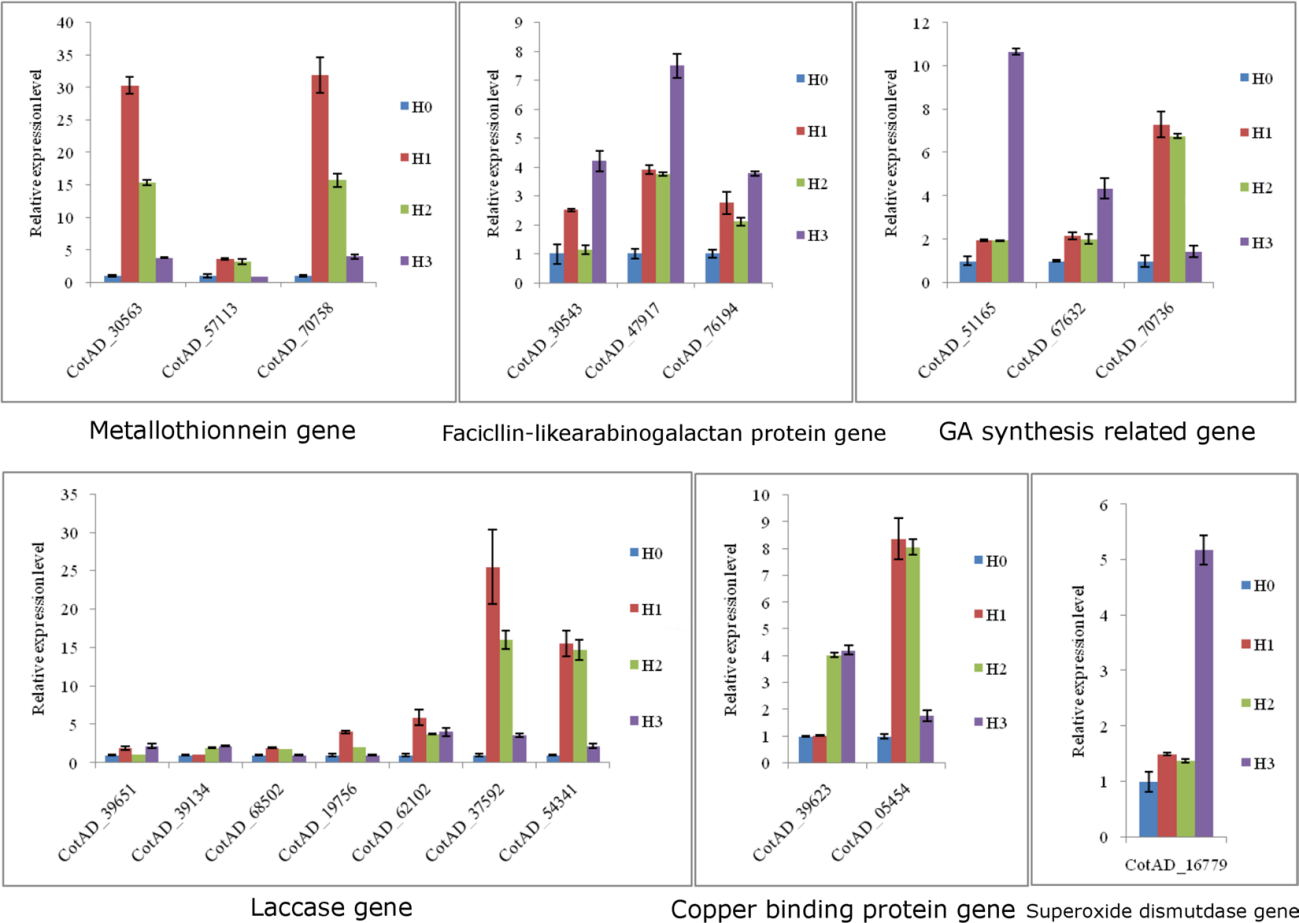
Verification of gene expression in untreated cotton seedlings (H0) and cotton seedlings treated with three different concentrations of cadmium (H1, H2 and H3) by real-time RT-PCR. (**A**) Metallothionein genes. (**B**) Facicllin-like arabinogalactan genes. (**C**) GA synthesis-related genes. (**D**) Laccase genes. (**E**) Copper binding protein genes. (**F**) Superoxide dismutase gene.

We performed real-time quantitative PCR to validate the expression of selected differentially expressed genes, including genes related to the formation of a physical barrier, anti-oxidization and chelation and root development. The expression levels determined by quantitative real-time PCR were basically consistent with the transcriptome data (Fig. 8). The expression level of FLA, which is a laccase involved in lignin synthesis and may be related to the sedimentation and thickening of the secondary cytoderm, increased under cadmium stress. The key anti-oxidative gene superoxide dismutase (SOD) (CotAD16779) and metallothionein (MT) genes that encode proteins that chelate heavy metal ions (CotAD30563, CotAD57113, CotAD70758) are all up-regulated, indicating an increase in the activity of antioxidant enzymes. Gibberellic acid synthetase, which is involved in root development was dramatically upregulated by cadmium stress. In a previous study, Gou *et al*. suggested that excessive amounts of gibberellin may suppress the development of the side roots of *Populus tremula*^62^. So, the accumulation of gibberellin may be one of the reasons why the development of cotton roots is impeded by cadmium stress.

## Conclusion

Transcriptome analysis of cotton roots exposed to cadmium stress revealed that physical barriers, including thichening of the cell wall and the casparian strip (CASP), may play an important role in the response to cadmium stress. In addition, an increase in pH in the root may enhance cadmium tolerance and also affect root development. Resistance to oxidation stress and detoxification via heavy metal complexation likely also play important roles. In addition, analysis of the functions of transcription factors differentially expressed in response to cadmium stress may have revealed a cotton-specific brassinolide (BR) signaling pathway that mediates the response cadmium stress. It was previously found that in *L. Chinense* response to cadmium stress is mediated by an endogenous salicylic acid signaling pathway, and overexpression of the Lycium chinense glutathione synthetase (LcGSHS) gene increased cadmium tolerance in transgenic *A. thaliana*^63^. The salicylic acid signaling pathway is often synergistic with the brassinolide (BR) signal pathway in environmental stress. Meanwhile, we found NPR1 which plays a key cross-talk between SA and brassinolide (BR) which via brassinolide (BR) signal activate the oxygen species (ROS) clearance mechanism was down-regulated signally under cadmium (Cd) stress in cotton^64,65^. So, it is may have a crosstalk between brassinolide (BR), ethylene signaling and cadmium (Cd) stress that help to improve cadmium (Cd) tolerance in cotton.

## Methods

### Plant Growth and Treatment

The C184 cotton species used for the experiment was provided by Hunan Cotton Science Institute. The cadmium solution was prepared with CdCl_2_·2.5H_2_O. The cotton seedlings were grown in Kimura B nutrient solution containing the following macronutrients^66^ (mM): (NH_4_)_2_SO_4_ (0.18), MgSO_4_·7H_2_O (0.27), KNO_3_ (0.09), Ca(NO_3_)_2_·4H_2_O (0.18), and KH_2_PO_4_ (0.09); and the following micronutrients (µM): MnCl_2_·4H_2_O (0.5), H_3_BO_3_ (3), (NH_4_)_6_Mo_7_O_24_·4H_2_O (1), ZnSO_4_·7H_2_O(0.4), Fe-EDTA (20), and 0.2 µM CuSO_4_·5H_2_O. The pH of the nutrient solution was adjusted to 5.5. Growth conditions were as follows: 27/24 °C day/night temperatures, 60–80% relative humidity, and a 14/10-h day/night photoperiod. The cultures were placed in an artificial intelligence (AI) climatic chamber for all-weather illumination. Four solutions containing different concentrations of CdCl_2_ added to the cultures when the seedlings developed two leaves and one core. The concentrations of the solutions were as follows: 0 mmol/L (H0, control), 1 mmol/L (H1), 5 mmol/L (H2) and 10 mmol/L (H3). Seedlings were grown for an additional 10 days (from October 12^th^ to October 21^st^). All root systems of seedlings from the four different treatments were taken out at the end of treatment, cleaned with tap water before rinsing with double distilled water, and then dried with sterile absorbent paper. The root tissues were then placed in a 5 ml germ-free centrifuge tube and then quickly frozen in liquid nitrogen, and submitted to the beijing genomics Institute (BGI) for sequencing analysis.

### Unigene annotation

Unigene annotations include functional annotation and information about expression. The comment information covers cluster of orthologous group (COG) functional annotation, protein functional annotation and gene ontology (GO) (http://geneontology.org/) functional annotation of unigenes based on BLASTX alignments to protein databases such as nr (http://www.ncbi.nlm.nih.gov/sites/entrez?db=protein), Swiss-Prot (http://www.expasy.org), KEGG (www.kegg.jp/kegg/kegg1.html) and COG (E-value < 0.00001) (http://www.ncbi.nlm.nih.gov/COG/). The proteins with the highest sequence similarity to unigenes are retrieved along with their protein and functional annotations. The gene ontology (GO) terms for upland cotton genes were obtained using the software Blast2GO (http://www.blast2go.com). At the same time, pathway analysis done using the KEGG mapping method. Enzyme commission numbers were assigned to unique sequences that had BLASTX scores with cutoff values of E < 1.0e5, as determined based on protein database searches. The Unigene sequences were mapped to the KEGG biochemical pathways according to the EC distribution in the pathway database.

### Quantitative RT-PCR

Total RNA was extracted from cotton root tissues using the Aidlab RNA extraction kit and was synthesized into cDNA using the Promega reverse transcription kit. The volumes of RNA from the four samples were adjusted with RNA-ase free H_2_O to ensure that the concentrations were the same before reverse transcription. Primer-BLAST was used to design the primers for fluorescent quantitation. RT-PCR was conducted on the ABI 7500 fast platform. Each reaction was done in a total volume of 20 µl, including 1 µl primer, 1 µl 10-fold diluted cDNA, 10 µl SYBR Green PCR mix, and brought up to volume with H_2_O. The cycling conditions were as follows: 95 °C 10 min followed by 95 °C 5 s, 60 °C 30 s, 72 °C 30 s, for a total of 40 cycles. The following conditions were used for melting curve analysis: 95 °C 15 s, 60 °C 1 min, 95 °C 30 s, 60 °C 15 s. Ghactin7 was used as the reference gene, and 2^−ΔΔCT^ was used to calculate the relative expression level^67^. Each reaction was repeated three times for each gene. The primer of Real-time fluorescent quantitative PCR see (supplementary material- primer).

### Enzyme activity assays

Superoxide dismutase enzyme and Thioredoxin peroxidase (TPx) enzyme activity was tested by kit of Sangon Biotech. Weighing 0.2 g root tissue and added to 0.8 ml phosphate buffer to 20% tissue homogenate for enzyme assays. Homogenates were spun at 4000 rpm/min in a centrifuge for 10 min. A volume of 0.1 ml supernatant was removed for superoxide dismutase enzyme activity determination, and double distilled water was used as the control. The samples were incubated in a water bath for 40 min and 37 °C, thermostatic and sufficient mixing, After adding 2 ml color developing reagent, samples were incubated 10 min at room temperature and then the absorbance at 550 nm of each sample was measured. The formula used to calculate superoxide dismutase activity is as follows:

$$\begin{array}{c}SOD(\frac{U}{g})=\frac{OD(control)-OD(text)}{OD(control)}\div50 \% \times \frac{V(reaction)}{V(sample)}/C({homogenate})\end{array}$$

A 0.1 ml aliquot of the same supernatant used for superoxide dismutase enzyme activity determination was used to assay for thioredoxin peroxidase (TPx) activity. thioredoxin peroxidase (TPx) decomposes H_2_O_2_ in the presence of a reducing agent, and we detemermined thioredoxin peroxidase (TPx) enzyme activity by measuring the rate of decrease in absorbance at 240 nm and subtracting the catalase (CAT) enzyme activity. The formulas used to calculate thioredoxin peroxidase (TPx) activity are as follows:

$${TPX}(\frac{{\rm{U}}}{g})=\frac{{\rm{\Delta }}A(gross)}{\varepsilon \times d}\times \frac{V(gross)\times v(reaction)}{V(sample)\times W(sample)\times T}\times P(dilution\,ratio)-{E}_{CAT}$$

$${{E}}_{{CAT}}(\frac{{\rm{U}}}{{\rm{g}}})=\frac{{\rm{\Delta }}A(gross)}{\varepsilon \times (light\,path)}\times \frac{V({gross})\times V(reaction)}{V(sample)\times W(sample)\times T}\times P(dilution\,ratio)$$

## Electronic supplementary material

Supplementary Dataset

## Data Availability

The datasets used and/or analysed during the current study are available from the corresponding author on reasonable request.
