# Supplementary material for: Analysis of potential strategies for cadmium stress tolerance revealed by transcriptome analysis of upland cotton
Source: Sci Rep. 2019 Jan 14;9:86. doi: 10.1038/s41598-018-36228-z (PMC6331580; doi:10.1038/s41598-018-36228-z)
Supplement: Supplementary file 1 — Supplementary Dataset [file 41598_2018_36228_MOESM1_ESM.docx]

**Analysis of potential strategies for cadmium stress tolerance revealed by transcriptome analysis of upland cotton**

**Haodong Chen^1†^, Yujun Li^1†^,** **Xiongfeng Ma^2^, Lishuang Guo^1^, Yunxin He^1^, Zhongying Ren^2^, Zhengcheng Kuang^1^, Xiling Zhang^2*^, Zhigang Zhang^1*^**

^1^Cotton Sciences Research Institute of Hunan/ National Hybrid Cotton Research Promotion Center, Changde, Hunan 415101, China, ^2^State Key Laboratory of Cotton Biology, Institute of Cotton Research, Chinese Academy of Agricultural Sciences, Anyang, Henan, 455000, China

**† Equal contributors.**

***** **Corresponding author.**

**Haodong Chen (†** **Equal contributors)**

Email: [chdmks@163.com](mailto:chdmks@163.com)

Address: Cotton Sciences Research Institute of Hunan/ National Hybrid Cotton Research Promotion Center, Hunan Changde 415101, China

**Yujun Li († Equal contributors)**

Email: 15974267467@163.com

Address: Cotton Sciences Research Institute of Hunan/ National Hybrid Cotton Research Promotion Center, Hunan Changde 415101, China

**Xiongfeng Ma**

Email:maxf_caas@163.com

Address: State Key Laboratory of Cotton Biology, Institute of Cotton Research, Chinese Academy of Agricultural Sciences, Anyang, Henan, 455000, China

**Lishuang** **Guo**

Email: mksgls@163.com

Address: Cotton Sciences Research Institute of Hunan/ National Hybrid Cotton Research Promotion Center, Hunan Changde 415101, China

**Yunxin He**

Email: heyunxin20@163.com

Address: Cotton Sciences Research Institute of Hunan/ National Hybrid Cotton Research Promotion Center, Hunan Changde 415101, China

**Zhongying Ren**

Email: renzhongyingcotton@163.com

Address: State Key Laboratory of Cotton Biology, Institute of Cotton Research, Chinese Academy of Agricultural Sciences, Anyang, Henan, 455000, China

**Zhengcheng Kuang**

Email: [hnsmks@163.com](mailto:hnsmks@163.com)

Address: Cotton Sciences Research Institute of Hunan/ National Hybrid Cotton Research Promotion Center, Hunan Changde 415101, China

**Xiling Zhang (*Corresponding author)**

Email: zhangxiling1962@126.com

Address: State Key Laboratory of Cotton Biology, Institute of Cotton Research, Chinese Academy of Agricultural Sciences, Anyang, Henan, 455000, China

**Zhigang Zhang (*Corresponding author)**

Email: zhangzhig@126.com

Address: Cotton Sciences Research Institute of Hunan/ National Hybrid Cotton Research Promotion Center, Hunan Changde 415101, China

The data of figure 4-A

| **Gene and Blast** | **log2Ratio(H0-VS-H1)** | **log2Ratio(H0-VS-H2)** | **log2Ratio(H0-VS-H3)** |
| --- | --- | --- | --- |
| CotAD_73900 Phenylalanine ammonia-lyase 2 | 1.502147 | 1.761501 | 1.374353 |
| CotAD_58842 Phenylalanine ammonia-lyase 2 | 1.461444 | 1.950942 | 1.437261 |
| CotAD_65518 Phenylalanine ammonia-lyase 2 | 1.476219 | 2.327277 | 1.639343 |
| CotAD_44197 PHE ammonia lyase 1 | 1.01598 | 1.263975 | 1.023476 |
| CotAD_76607 PHE ammonia lyase 1 | 1.196994 | 1.419636 | 1.010774 |
| CotAD_20123 cinnamate-4-hydroxylase | 1.119793 | 1.706111 | 1.290829 |
| CotAD_35147 cinnamate-4-hydroxylase | 1.250456 | 1.857079 | 1.370431 |
| CotAD_58418 cinnamate-4-hydroxylase | 1.619565 | 2.355231 | 1.621664 |
| CotAD_68335 4-coumarate:CoA ligase 1 | 1.232104 | 1.659913 | 1.294002 |
| CotAD_24031 4-coumarate:CoA ligase 1 | 1.037868 | 2.121872 | 1.972888 |
| CotAD_09749 4-coumarate:CoA ligase 2 | 1.901457 | 1.970203 | 1.672213 |
| CotAD_31895 4-coumarate:CoA ligase 2 | 1.425355 | 2.624677 | 2.23906 |
| CotAD_26997 4-coumarate:CoA ligase 2 | 1.332961 | 1.376268 | 1.524786 |
| CotAD_52480 4-coumarate:CoA ligase 2 | 1.317443 | 1.596568 | 1.443526 |
| CotAD_23412 4-coumarate:CoA ligase 2 | 1.165909 | 1.620308 | 1.443103 |
| CotAD_28319 p-coumarate 3-hydroxylase | 3.460432 | 3.563698 | 3.222786 |
| CotAD_03771 ferulate 5-hydroxylase | 2.062508 | 2.39945 | 2.682611 |
| CotAD_37261 ferulate 5-hydroxylase | 1.629531 | 2.313472 | 2.600411 |
| CotAD_33040 CoA O-methyltransferase 1 | 1.824204 | 1.456113 | 2.175636 |
| CotAD_33041 CoA O-methyltransferase 1 | 1.824204 | 1.456113 | 2.175636 |
| CotAD_04328 CoA O-methyltransferase 1 | 1.27674 | 1.451352 | 1.657855 |
| CotAD_14339 CoA O-methyltransferase | 1.495515 | 1.302892 | 2.082444 |
| CotAD_46258 CoA O-methyltransferase 2 | 2.061509 | 2.495334 | 2.98591 |
| CotAD_13393 Peroxidase superfamily protein | 4.438533 | 4.079289 | 4.065237 |
| CotAD_24672 Peroxidase superfamily protein | 3.889848 | 4.029508 | 4.221764 |
| CotAD_12958 Peroxidase superfamily protein | 3.699544 | 3.631705 | 4.434628 |
| CotAD_48772 Peroxidase superfamily protein | 3.944858 | 3.766713 | 4.423279 |
| CotAD_13112 Peroxidase superfamily protein | 4.633489 | 5.000556 | 5.477733 |
| CotAD_72461 Peroxidase superfamily protein | 5.306259 | 5.535707 | 5.830388 |
| CotAD_44491 Peroxidase superfamily protein | 5.446361 | 5.308122 | 4.527539 |
| CotAD_40570 Peroxidase superfamily protein | 2.392317 | 2.779341 | 3.062773 |
| CotAD_16807 Peroxidase superfamily protein | 2.171648 | 2.154328 | 3.541659 |
| CotAD_15698 Peroxidase superfamily protein | 2.276713 | 2.540254 | 3.377686 |
| CotAD_45711 Peroxidase superfamily protein | 3.090437 | 2.757697 | 2.540519 |
| CotAD_12956 Peroxidase superfamily protein | 3.15313 | 4.23362 | 2.942515 |
| CotAD_43905 Peroxidase superfamily protein | 3.279389 | 3.7381 | 4.208225 |
| CotAD_37264 Peroxidase superfamily protein | 2.214031 | 1.809931 | 2.796485 |
| CotAD_61438 Peroxidase superfamily protein | 1.821258 | 2.266097 | 2.857049 |
| CotAD_12955 Peroxidase superfamily protein | 1.82971 | 1.95099 | 2.337035 |
| CotAD_35621 Peroxidase superfamily protein | 1.412425 | 1.481353 | 1.8829 |
| CotAD_55319 Peroxidase superfamily protein | 1.571187 | 1.676885 | 1.786413 |
| CotAD_13466 Peroxidase superfamily protein | 1.808271 | 1.59136 | 1.856875 |
| CotAD_39461 Peroxidase superfamily protein | 5.031586 | 3.523562 | 5.467606 |
| CotAD_15696 class III peroxidase | 3.966392 | 4.341322 | 4.894909 |
| CotAD_61437 class III peroxidase | 2.567475 | 2.694813 | 3.195955 |
| CotAD_61436 class III peroxidase | 2.468873 | 2.871439 | 3.328359 |
| CotAD_33573 class III peroxidase | 1.059997 | 1.14991 | 1.591376 |
| CotAD_54288 class III peroxidase | 1.592636 | 1.539366 | 1.944109 |
| CotAD_15694 class III peroxidase | 6.280931 | 6.125013 | 7.085633 |
| CotAD_28413 bacterial-induced class III peroxidase | 1.73034 | 1.965567 | 2.209558 |
| CotAD_02264 Peroxidase 64 | 1.813379 | 2.360053 | 1.995466 |
| CotAD_43126 Peroxidase 24 | 2.616591 | 1.801393 | 2.098097 |
| CotAD_16071 Peroxidase 68 | 2.803728 | 3.194449 | 2.389094 |
| CotAD_73087 Peroxidase 24 | 3.792763 | 2.862753 | 1.944883 |
| CotAD_40407 Peroxidase 68 | 3.29154 | 3.092027 | 3.364212 |
| CotAD_51945 Laccase 12 | 10.216746 | 11.192293 | 10.85331 |
| CotAD_30570 Laccase 17 | 4.343954 | 4.332983 | 4.70044 |
| CotAD_08665 Laccase 3 | 4.701909 | 4.461601 | 4.413544 |
| CotAD_03385 Laccase 17 | 2.73588 | 2.743999 | 2.52968 |
| CotAD_74213 Laccase 17 | 2.600695 | 2.337035 | 2.559427 |
| CotAD_51939 Laccase 12 | 1.324435 | 2.231326 | 1.707179 |
| CotAD_22333 Laccase 12 | 1.321928 | 2.203583 | 1.391961 |
| CotAD_74215 Laccase 17 | 1.672582 | 1.977912 | 1.790173 |
| CotAD_39674 Diphenol oxidase family protein | 2.646739 | 2.1127 | 2.004501 |
| CotAD_56925 Diphenol oxidase family protein | 2.1133 | 2.323813 | 2.413268 |
| CotAD_40519 Diphenol oxidase family protein | 1.592761 | 2.571213 | 2.65181 |
| CotAD_29825 Diphenol oxidase family protein | 1.369546 | 2.463826 | 2.79066 |
| CotAD_37592 Diphenol oxidase family protein | 1.816491 | 1.855767 | 1.551761 |
| CotAD_62102 Diphenol oxidase family protein | 1.786366 | 1.864192 | 1.560201 |
| CotAD_09457 Diphenol oxidase family protein | 1.724668 | 1.557923 | 1.415994 |
| CotAD_30582 Diphenol oxidase family protein | 1.313228 | 1.382509 | 1.430494 |
| CotAD_44630 Casparian strip membrane protein | 13.790552 | 12.486835 | 14.34776 |
| CotAD_70754 Casparian strip membrane protein | 4.609061 | 3.988443 | 4.751473 |
| CotAD_67444 Casparian strip membrane protein 1 | 6.078502 | 5.73364 | 5.978485 |
| CotAD_19116 Casparian strip membrane protein 3 | 12.382624 | 13.246741 | 13.647234 |
| CotAD_26673 Casparian strip membrane protein 3 | 5.260026 | 4.3084 | 4.83727 |
| CotAD_26672 Casparian strip membrane protein 3 | 5.309855 | 5.073682 | 5.591928 |
| CotAD_19112 Casparian strip membrane protein 3 | 4.1325 | 3.563673 | 4.127672 |
| CotAD_30282 Casparian strip membrane protein 3 | 3.168219 | 3.406861 | 2.607802 |
| CotAD_34676 Casparian strip membrane protein 5 | 5.861933 | 5.59856 | 4.573991 |

The data of figure 5-A

| **Gene and Blast** | **log2Ratio(H0-VS-H1)** | **log2Ratio(H0-VS-H2)** | **log2Ratio(H0-VS-H3)** |
| --- | --- | --- | --- |
| CotAD_64954 Thioredoxin-dependent peroxidase 1 | 3.388576 | 3.773325 | 3.549313 |
| CotAD_09621 Thioredoxin-dependent peroxidase 2 | 4.050085 | 3.296617 | 3.759269 |
| CotAD_02283 Ascorbate peroxidase | 1.202448 | 1.51981 | 1.159478 |
| CotAD_67897 microsomal glutathione s-transferase | 2.041838 | 1.626503 | 2.205585 |
| CotAD_41052 microsomal glutathione s-transferase | 2.467954 | 1.493492 | 2.007563 |
| CotAD_11525 Tau class glutathione transferase GSTU43 | 2.208355 | 2.848138 | 3.073953 |
| CotAD_30500 Tau class glutathione transferase GSTU43 | 1.48763 | 1.992034 | 2.416966 |
| CotAD_11529 Tau class glutathione transferase GSTU43 | 1.559411 | 2.081291 | 2.397383 |
| CotAD_43155 Glutathione S-transferase family protein | 1.051187 | 1.795842 | 1.749814 |
| CotAD_11531 Glutathione S-transferase family protein | 2.007898 | 2.219025 | 2.983207 |
| CotAD_30563 metallothionein-like protein | 2.411633 | 2.717162 | 2.502126 |
| CotAD_70758 metallothionein-like protein | 2.928321 | 3.277581 | 3.262847 |
| CotAD_03497 metallothionein-like protein | 1.194791 | 1.521234 | 1.308351 |
| CotAD_57113 metallothionein 3 | 1.465979 | 1.135531 | 1.219314 |
| CotAD_16779 Superoxide dismutase | 1.919791 | 1.446547 | 1.203917 |
| CotAD_70179 Plant PDR ABC-type transporter family protein | 3.282316 | 3.238405 | 3.884679 |
| CotAD_61730 Plant PDR ABC-type transporter family protein | 1.640997 | 1.841634 | 2.284303 |
| CotAD_30564 Non-intrinsic ABC protein 9 | 1.404903 | 1.290424 | 2.039138 |
| CotAD_14629 ZIP metal ion transporter family | 1.20792 | 1.892115 | 1.493952 |
| CotAD_54912 copper binding protein 1 | 1.201196 | 1.366078 | 1.159027 |
| CotAD_39623 copper binding protein 3 | 2.754603 | 2.543441 | 2.144139 |
| CotAD_26399 copper binding protein 3 | 2.478891 | 2.296397 | 2.127383 |
| CotAD_26404 copper binding protein 3 | 2.478891 | 2.296397 | 2.127383 |
| CotAD_05454 copper binding protein 4 | 1.423316 | 1.384795 | 1.67188 |
| CotAD_10158 copper binding protein 4 | 1.806316 | 2.555285 | 2.62763 |
| CotAD_10313 copper binding protein 7 | 2.589561 | 2.443769 | 1.817584 |
| CotAD_48170 Cadmium/zinc-transporting ATPase 3 | 1.30221 | 1.139875 | 1.34138 |
| CotAD_73136 Vacuolar membrane ATPase 10 | 1.205267 | 1.480483 | 1.788029 |
| CotAD_59705 ATP synthase delta subunit 1 | 1.156465 | 1.450246 | 1.386021 |
| CotAD_30126 MATE efflux family protein | 3.873987 | 4.35962 | 4.222014 |

The primer of Real-time fluorescent quantitative PCR

| **Primer name** | **Primer bases** |
| --- | --- |
| Actin-F | ATCCTCCGTCTTGACCTTG |
| Actin-R | TGTCCGTCAGGCAACTCAT |
| CotAD_30563-F | TGCTGTTCAGGAAAATGTGGCT |
| CotAD_30563-R | ATCCACCCTCAACCCCAACTC |
| CotAD_57113-UP | CGTGAAGAAGGGAAGCAGTAACAC |
| CotAD_57113-Dn | TTAACGACCACATGTGCAGTTG |
| CotAD_70758-UP | CTGTGGAGGATGCAAGATGTTC |
| CotAD_70758-Dn | TCTCAACCCCAACTCCCATCTC |
| CotAD_30543-UP | GGTTCAGTTCCATATACTCCCG |
| CotAD_30543-Dn | GGTCGTCACATTCACTTGGTTC |
| CotAD_47917-UP | TACCGATATACCTCCAACGCCT |
| CotAD_47917-Dn | CAACCCACCTAAGACTGCCAA |
| CotAD_76194-UP | GTATTGCTGTTGTACTCCTCCTTG |
| CotAD_76194-Dn | CAGTGTTGTTGGCTTGGTTTTG |
| CotAD_51165-UP | GCTTCACTTCTCATCTCTCTTCTC |
| CotAD_51165-Dn | ACACATTTCTTGGTTACCGG |
| CotAD_67632-UP | TGGAGTAATGGGAGATTGAAAGC |
| CotAD_67632-Dn | TTGGTGGTGAGAGAGAAGCAA |
| CotAD_70736-UP | GTTGCTACTTTCTTCTTGGCTC |
| CotAD_70736-Dn | TGGTTTATGGTACTGTGTCTGG |
| CotAD_39651-UP | ACTCTCATACCTCCACGCTCCG |
| CotAD_39651-Dn | CGACATTACCATTCCACCACTCA |
| CotAD_39134-UP | GAGCTACCTATACAACTTCACCCT |
| CotAD_39134-Dn | GGAAATGGGTAAGGAACACC |
| CotAD_68502-UP | ATGGCAGGATGGAGTGTTGG |
| CotAD_68502-Dn | TGCATAAGGAATTGGGATCACAC |
| CotAD_19756-UP | CGAAATCACACGGCTTTGAGAA |
| CotAD_19756-Dn | TCTGCTGCACCGTATATGATCC |
| CotAD_62102-UP | TCCACCATTCACCTAACACAACAA |
| CotAD_62102-Dn | TCCACCATTCACCTAACACAACAA |
| CotAD_37592-UP | GGATTAGGCAAATACGTACG |
| CotAD_37592-Dn | GGAAAGGATAAGGAACATCAC |
| CotAD_54341-UP | AGTTGAAGTTGGTTGAAGTGGAG |
| CotAD_54341-Dn | AGAGTTGCTGTTGAGGTGAGAAC |
| CotAD_39623-UP | TGGACCACGACCGGAGATTA |
| CotAD_39623-Dn | CTGGATTGGACAGTGTGATGACA |
| CotAD_05454-UP | GCTGTCGGATTGGCTTTAGTGT |
| CotAD_05454-Dn | CAGTTGTGAAGAGGATGATCAGCA |
| CotAD_16779-UP | TATTAGTGAAGGAGGAGGTGAGC |
| CotAD_16779-Dn | CCAAACATCTATCCCAAGCAA |
